# Spontaneous corneal melting during pregnancy: a case report

**DOI:** 10.1186/1757-1626-2-7444

**Published:** 2009-05-26

**Authors:** Joo Youn Oh, Mee Kum Kim, Joong Shin Park, Won Ryang Wee

**Affiliations:** 1 Department of Ophthalmology, Seoul National University College of MedicineSeoulKorea; 2 Department of Obstetrics & Gynecology, Seoul National University College of MedicineSeoulKorea

## Abstract

**Introduction:**

Biomechanical changes in the cornea during pregnancy might lead to pathological conditions such as corneal perforation or melting.

**Case presentation:**

A 33-year-old Asian female who underwent penetrating keratoplasty in both eyes developed corneal melting in the right eye and marginal keratitis in the left eye in her fifth month of pregnancy. Marginal keratitis in the left eye immediately subsided with topical steroid therapy. However, spontaneous corneal melting progressed in the right eye, despite oral steroid therapy and amniotic membrane transplantation. We performed tectonic penetrating keratoplasty and corneoscleral grafting in the right eye.

**Conclusion:**

We advise caution in the ophthalmologic care of pregnant patients who have preexisting corneal thinning disorders or who have undergone multiple corneal surgeries, because physiologic changes during pregnancy might contribute to corneal changes leading to spontaneous melting especially in patients with compromised cornea.

## Introduction

Hormonal and immunological changes that occur during pregnancy affect all tissues, including the eye [1]. Pregnancy induces physiological changes in the cornea, including an increase in corneal thickness and curvature and decrease in corneal sensitivity [1]. However, several case reports have demonstrated that biomechanical changes in the cornea during pregnancy might lead to pathological conditions such as corneal perforation or melting [2,3]. We report a case of a woman who developed spontaneous corneal melting which started in her fifth month of pregnancy, and required tectonic corneal grafting.

## Case presentation

A 33-year-old Asian woman in her fifth month of pregnancy presented with discomfort and redness in her left eye. The patient had history of penetrating keratoplasty in both eyes secondary to corneal melting of unknown cause 15 years previously. She had been on the prolonged self-use of steroid eye drops at that time. Despite extensive screening, no immune-mediated or infectious condition was identified. The patient was not pregnant at that time. Thereafter, she had recurrent episodes of corneal melting in the right eye and underwent two more penetrating keratoplasties and one corneoscleral grafting procedure in the right eye. Every episode was accompanied by spontaneous corneal melting without obvious inflammatory signs. She remained asymptomatic for the next ten years, until her fifth month of her first pregnancy. She had no history of ocular trauma or systemic disease, and her pregnancy was uncomplicated.

**Figure 1.**
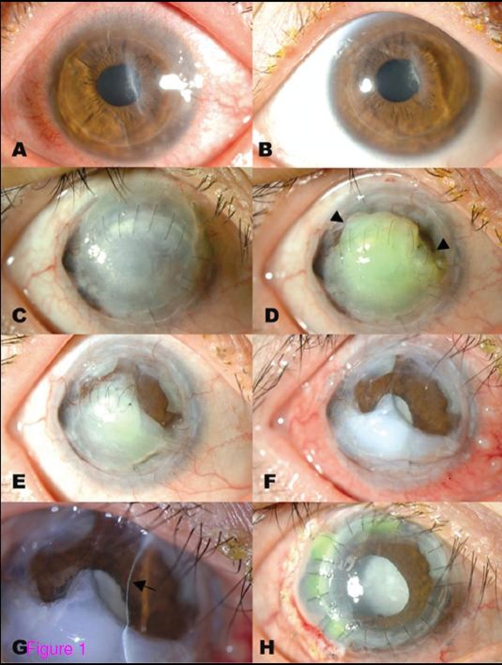
Slit lamp appearance of both eyes of a patient in the midterm of pregnancy.

Examination revealed several round infiltrations in the peripheral cornea in the left eye, accompanied by limbal edema and conjunctival injection (Figure 1A). Her right eye remained stable (Figure 1C). We prescribed topical prednisolone eye drops four times per day for her left eye. Two weeks later, the inflammatory signs and symptoms in her left eye completely subsided (Figure 1B). Meanwhile, her right eye developed corneal melting beginning in the superior part of the cornea (Figures 1D, 1E). No inflammatory signs or symptoms, such as pain, conjunctival injection, or corneal infiltrates, were observed. Systemic screening for rheumatologic diseases was performed; all test results were negative. Schirmer test revealed normal tear production in both eyes, and her lid function was normal. We prescribed oral prednisolone 30 mg per day after consultation with an obstetrician, and we performed amniotic membrane transplantation. Despite these efforts, the spontaneous corneal melting progressed, and Descemet's membrane became visible in the superior half of the cornea (Figures 1F, 1G). In the patient's seventh month of pregnancy, we performed penetrating keratoplasty in the right eye for tectonic purposes (Figure 1H). Corneoscleral grafting was also performed for the extremely thinned part of the peripheral cornea.

The left eye presented with diffuse conjunctival injection and multiple, round infiltration in the inferotemporal periphery of cornea, suggesting marginal keratitis (A). The inflammation was completely resolved after two weeks of topical prednisolone therapy (B). Meanwhile, the right eye, which was totally opaque but remained stable for ten years after having undergone three penetrating keratoplasties (C), developed corneal graft melting without inflammation signs and symptoms (D, arrowhead). The graft lysis progressed over the next two months (E). Amniotic membrane transplantation was performed to stop the lysis of corneal graft, which failed (F). Descemet's membrane was visible in the superior half of the cornea (G, arrow). Eventually, the full-thickness corneal transplantation with tectonic corneoscleral grafting was performed (H).

## Discussion

Pregnancy produces a large number of immunological and hormonal changes that affect the cornea [1]. As for pathological changes in the cornea, two reports have previously described spontaneous corneal melting or perforation occurring during the late phase of pregnancy (5-9 months). One was a case of spontaneous corneal melting in a woman during her seventh month of pregnancy [2]. She had undergone radial keratotomy nine years previously. Another was a case of corneal perforation in a patient with preexisting keratoconus [3]. Marked corneal edema and perforation occurred in the eighth month of pregnancy, and transient iritis preceded corneal perforation. Our patient was in her fifth month of pregnancy when she developed transient marginal keratitis in one eye and spontaneous corneal melting in the other eye, which progressed to the point that it required a tectonic corneal graft. There were no obvious inflammatory signs or symptoms in the eye with corneal melting.

These phenomena are possibly two-fold: hormonal and immunological. A host of hormonal changes occur during pregnancy and have different effects on various parts of the body. Estrogen, which markedly increases in the midterm of pregnancy (5-9 months), is known to affect the biomechanical stability of the cornea by producing matrix metalloproteinases and causing prostaglandin release, thereby activating collagenases [4-6]. This stiffness-reducing effect of estrogen on the cornea may be responsible for the higher risk of regression or keratectasia after refractive surgery in pregnant women or those on hormone replacement therapy [7-10]. In a normal pregnancy, this biomechanical effect of estrogen is widely compensated by progesterone, because progesterone suppresses the production of collagenase and prostaglandin [11]. However, an estrogen-induced collagen network-weakening effect on the cornea might be exacerbated in a biomechanically weakened cornea such as is seen in the setting of keratoconus or after radial keratectomy or penetrating keratoplasty. Another important hormone produced during pregnancy is relaxin, which is a positive regulator of matrix metalloproteinase during gestation. This hormone has different effects on various collagen-containing organs [12]. Relaxin might exhibit collagenolytic properties in the cornea, especially compromised ones. This might lead to corneal melting, as was seen in our case. Furthermore, pregnancy is considered to be a state of relative immune suppression directed toward avoiding rejection of the semi-allogeneic fetus. However, the maternal immune system must remain immunocompetent to fight infections and to clear abnormal precancerous cells. Thus, most autoimmune diseases do not improve during pregnancy, while some autoimmune diseases, such as rheumatoid arthritis and multiple sclerosis, have been shown to be temporarily subside [13]. Regarding the eye, autoimmune uveitis has been shown to resolve during pregnancy [14], but allograft rejection still occurs [15]. We observed marginal keratitis in the patient in the present study, but this immediately responded to topical steroids. Likewise, the spontaneous corneal melting observed in the other eye of our patient might have been the result of either recurrence of sub-clinical corneal inflammation or the immune rejection followed by corneal melting rather than the result of spontaneous corneal collagenolysis secondary to internal hormonal changes.

## Conclusions

In summary, although the underlying mechanisms remain to be clarified, the patient in this study demonstrated that physiological changes during pregnancy might lead to devastating corneal complications, such as corneal melting, in high-risk women with compromised corneas. We advise caution in the ophthalmologic care of pregnant patients who have preexisting corneal thinning disorders or who have undergone corneal surgeries, even if they were performed many years previously.

## Patient's perspective

I had suffered from corneal melting 15 years ago. After that, there were no episodes of corneal melting. However, during my first pregnancy, I have had corneal melting without neither injection nor pain in my eye. Because of successful corneal transplant, I can save my eyeball. Now I gave birth to my baby safely, and my eye is stable. But I am afraid of having another baby because I am afraid I may have corneal melting during pregnancy again.

## Authors' information

OJY is an ophthalmologist and cornea specialist at Seoul national university hospital. PJS is an obstetrician and professor at Seoul national university hospital. WWR is an ophthalmologist, and chairman and professor at Seoul national university hospital.
